# The language context effect in facial expressions processing and its mandatory characteristic

**DOI:** 10.1038/s41598-019-47075-x

**Published:** 2019-07-30

**Authors:** Shen Liu, Qun Tan, Shangfeng Han, Wanyue Li, Xiujuan Wang, Yetong Gan, Qiang Xu, Xiaochu Zhang, Lin Zhang

**Affiliations:** 1 Department and Institute of Psychology, Ningbo University, Ningbo, China; 2 School of Humanities and Social Sciences, University of Science and Technology of China, Hefei, China; 3 Heze University, Heze, China; 4 College of Psychology and Sociology, Shenzhen University, Shenzhen, China; 5 CAS Key Laboratory of Brain Function and Disease, and School of Life Sciences, University of Science and Technology of China, Hefei, China; 6 Hefei Medical Research Center on Alcohol Addiction, Anhui Mental Health Center, Hefei, China; 7 Academy of Psychology and Behavior, Tianjin Normal University, Tianjin, China

**Keywords:** Perception, Human behaviour

## Abstract

Background visual scenes in which faces are perceived provide contextual information for facial expression processing. One type of background information, the language context, has a vital influence on facial expression processing. The current study is aimed to investigate the effect of the language context on facial expression processing by recording event-related potentials (ERPs). Experiment one adopted the facial expression categorization task to investigate the effects of different language contexts on emotional and non-emotional facial processing. Experiment two adopted the task-irrelevant paradigm to investigate whether the language context effect on facial expression processing was mandatory. The results found that (1) the language context affected facial expression processing. Facial expression processing was promoted when the language context was emotionally congruent with faces. Moreover, the language context had an evoking effect on neutral faces. To be detailed, neutral facial expressions were evoked to be judged as positive in the positive language context while as negative in the negative language context. (2) The language context effect still affected facial expression processing in a task-irrelevant paradigm. When the language context was emotionally incongruent with facial expressions, larger N170 and LPP amplitudes were elicited, indicating the inhibition of incongruent emotions. These findings prove that the language context effect on facial expression processing is mandatory.

## Introduction

Facial expressions are fundamental emotion stimuli because they convey important information in social interactions. In daily life, faces are embedded in surrounding contexts. It has been well-documented that faces are better remembered when displayed in the presence of the original learning context. The effect of either consistent or changing context on the ability to recall or recognize acquired information is known as the context effect^1^. As a type of background information, the situation described by language and text, that is, the language context, also has an important influence on facial expression processing, namely, the language context effect^2^. Thus, it is important to understand how the language context affects facial expression processing.

When the language context and faces are emotionally congruent, individuals’ preferences for faces are higher^3^. It indicates that the language context can not only influence individuals’ emotionally consistent judgment on facial expressions through emotional initiation, but also induce individuals’ expectation of facial expressions through semantic initiation, and prompt individuals to carry out deeper cognitive processing on facial expressions^3^. In addition, Diéguez-Risco *et al*. respectively used the classic facial expression categorization task and the emotional consistency judgment task to investigate the language context effect on happy and angry facial processing. They have found that the emotionally congruent language context and facial emotions affected both the early coding stage of face processing—to elicit a larger N170 amplitude—and emotional evaluations in the later stage of facial processing to elicit a larger LPP (late positive potentials) amplitude under the conflict condition^4^. Since the language context can be used as a carrier of semantic information, it can induce individuals’ emotional states to affect their subsequent processing of faces, along with other background factors^5^. However, previous studies investigating the language context effect on emotional facial processing do not distinguish between positive and negative language contexts well. Moreover, the emotional valence and arousal of the neutral language context are different from that of positive and negative language contexts. It is not suitable to investigate the different effects of various types of the emotional language context on emotional facial processing without the neutral language context.

There are three conclusions drawn on the effect of background information on facial expressions. Firstly, the promoting effect of emotional congruence. When facial expressions are embedded in the emotional background, they are influenced by its emotional content, which is reflected in the promotion of facial expression processing when both the background and faces are emotionally congruent^6,7^. Secondly, the inhibiting effect of emotional incongruence. Background information can not only facilitate facial expression processing, but also hinder it. Furthermore, this hindrance is mainly manifested in the processing interference of the target emotional face when the background information and facial expressions are emotionally incongruent^8^. Thirdly, the evoking effect of emotions. The effect of background information on facial expression processing is also manifested in the emotional initiation or induction of neutral faces. For example, Morel *et al*. have found that neutral facial processing with different sound backgrounds showed significant differences in brain activity at the early stage when sound backgrounds expressed different types of emotions, such as “he passed all his exams”^9^. Schwarz *et al*. also have found that neutral faces shown in a positive context were evaluated more positively than those shown in a negative context, and that this effect was more pronounced in a self-related context. In addition, neutral faces shown in a self-related context could elicit stronger activities in the medial prefrontal cortex and right fusiform gyrus^10^. Wieser *et al*. have found that the self-related language context and emotional contextual information could enhance both early and late stage EEG activities in neutral facial processing, and neutral faces appearing after the negative language context could elicit greater EPN (early posterior negativity)^11^. Those findings showed that emotional background information could evoke neutral faces to produce similar emotions to the background, which were reflected in corresponding neurophysiological activities. However, there are large differences in indifferent background information processing^12^, and a study has yet to be published on the language context effect. Thus, it is necessary to further investigate the specific effects of the language context on facial expression processing to determine the mechanism of the language context effect. Therefore, the current study proposed a hypothesis: the language context influences facial expression processing. To be specific, the language context effect on facial expression processing had a promoting effect if the RTs for judging facial expressions when expressions and language contexts were emotionally congruent were significantly shorter than that of irrelevant and incongruent while there was no significant difference between irrelevant and incongruent, an inhibiting effect if the RTs for judging facial expressions when expressions and language contexts were emotionally incongruent were significantly longer than that of irrelevant and congruent while there was no significant difference between irrelevant and incongruent, and an evoking effect on non-emotional faces (neutral faces).

Previous studies have investigated the processing characteristics of the context effect and found that some background factors such as scene pictures, body posture, and sound have an automatic effect on facial expression processing^13,14^. However, whether the integration of the language context and facial expressions is an automated or a controlled process is still unclear. In real interpersonal interactions, it is not conducive to reveal the language context effect on facial expression processing, nor is it conducive to assess the importance of the language context on facial expressions. To fully reveal the processing characteristics of the background effect and analyze the differences between different emotional backgrounds’ effects on facial expression processing, it is necessary to use the language context as the background information to deeply explore its effect on facial expression processing, as well as its processing characteristics. Different facets of automaticity have been set out, such that automatic process can be rapid, non-conscious, mandatory, or capacity-free^15–17^. Here, we ask whether the language context effect on facial expression processing is mandatory, i.e., whether it occurs regardless of one’s intention, which is the most important characteristic of the automated processing^15,18^. Neath-Tavares and Itier have found that the N170 amplitude elicited by facial expressions presented above the scene picture was moderated by different emotional scenes^19^. It indicated that the context effect of facial expression processing could be unintentional. Yang *et al*. also found similar results; in the face gender judgment task, the early perceptual coding of facial expressions was moderated by the emotions of the scene, demonstrating the unintentional effect of the scene^14^. However, previous studies involving unintentionality use facial pictures, but not language contexts like sentences. Language contexts, including sentences involving semantic processing, and previous studies found that semantic processing had unintentionality^20,21^. Therefore, the current study proposed a second hypothesis: the language context effect on facial expression processing is mandatory. To be specific, the facial expression processing in gender judgment tasks was still influenced by the language context.

In summary, although existing studies have revealed the language context effect on facial expression processing^3,9^, there are still some issues to discuss further. On the one hand, previous studies on the neural mechanism of the language context effect are not comprehensive^12^. The language context effect of different types of emotions on facial expression processing, and whether there are differences of the same type of the emotional language context on the processing between emotional and non-emotional faces are not fully discussed. On the other hand, whether the language context effect on facial expression processing is an automated cognitive process, the way other scene effects are, is still unknown^6,19^. It is still inconclusive as to whether the automatic integration of the background information and facial expression is only at the perceptual processing level or if also involves deeper semantic processing. However, background information must break through the limitations of individual consciousness and cognitive resources in order to automatically integrate the processing of facial emotions, helping individuals process the emotional information found in facial muscle movements more quickly and accurately, which has important practical applications for human survival and adaptation. The current study designed two experiments. Experiment one investigated the language context effect on facial expression processing using classical expression category judgment tasks. Experiment two investigated the processing characteristics of the language context effect on facial expression processing using the task-irrelevant paradigm (gender judgment task) to learn whether the language context effect on facial expression processing also had automated processing characteristics.

## Experiment 1

### Methods

#### Participants

Thirty-three right-handed college students (29 females) with a mean age of 21.26 (*SD* = 1.39) were paid to participate in the experiment. After receiving a complete description of the study, all volunteers gave written informed consent. Participants had no prior history of neurological or psychiatric problems and had normal or corrected-to-normal vision. They were paid a fixed amount (50 RMB) for their participation. The current study was approved by the Ethics Committee of Ningbo University in accordance with the ethical principles of the Declaration of Helsinki.

#### Experimental design

A 3 (types of the language context: positive, neutral, negative) × 3 (types of facial expressions: positive, neutral, negative) within-subject design was adopted. The dependent variables were the response times (RTs) and accuracy rates (ACCs) for judging the types of facial expressions.

#### Stimuli

Sixty face pictures including 20 happy, 20 neutral and 20 sad expressions were obtained from the native Chinese Facial Affective Picture System (CFAPS)^22^. These images were unfamiliar to the participants, and included no film stars, well-known musicians, or other celebrities. All faces were with a frontal view and forward eye-gaze. The photos were cropped to remove hair and ears, leaving only a facial mask. All stimuli were converted into grayscale images with dimensions of 202 × 225 pixels on a black background. They were matched with each other in size, brightness, contrast grade and attractiveness.

We created 120 contextual sentences, of which 40 positive (e.g., “This person just picked up ten thousand RMB”), 40 neutral (e.g., “This person just opened the window”) and 40 negative (“This person just broke up with his/her partner”) sentences were collected. These sentences were 9~11 words in length and used only familiar words. A pilot study with 33 participants evaluated these contextual sentences on valence (from 1 = very negative to 9 = very positive) and arousal (from 1 = calm to 9 = extremely arousing) on a 9-point scale. Finally, thirty positive, thirty neutral, and thirty negative contextual sentences were selected. A single factor ANOVA was used to analyze the valence and arousal of three types of contextual sentences and the results were as follows. There was a significant difference of the valence of the three types of contextual sentences (*F*_(2,31)_ = 915.83, *p* < 0.001, η^2^_*p*_ = 0.98) indicating that the valence of positive contextual sentences (*M* = 6.98, *SD* = 0.37) was significantly larger than that of neutral (*M* = 5.32, *SD* = 0.19; *p* < 0.01) and negative (*M* = 2.79, *SD* = 0.33; *p* < 0.01) contextual sentences, and the valence of neutral contextual sentences was significantly larger than that of negative contextual sentences (*p* < 0.001). There was also a significant difference of the arousal of the three types of contextual sentences (*F*_(2,31)_ = 519.40, *p* < 0.001, η^2^_*p*_ = 0.97) indicating that the arousal of positive (*M* = 6.25, *SD* = 0.46; *p* < 0.001) and negative (*M* = 6.00, *SD* = 0.57; *p* < 0.001) contextual sentences was significantly larger than that of neutral contextual sentences (*M* = 4.54, *SD* = 0.21) while there was no significant difference between positive and negative contextual sentences (*p* = 0.097).

#### Procedure

**Figure 1:**
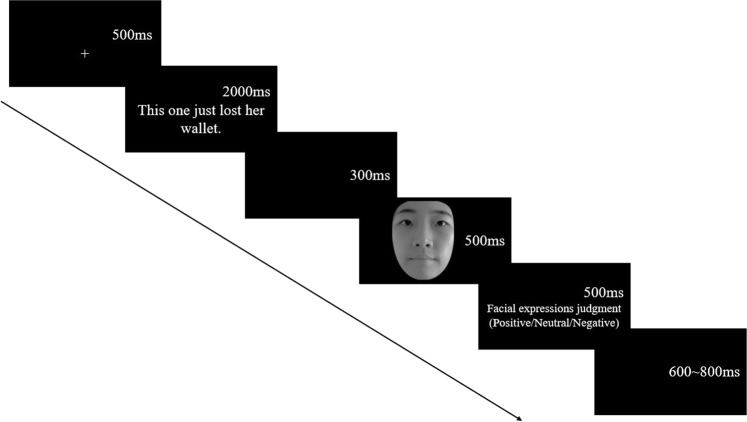
Timings and displays of one trial in the task. (Please reader note that due to the privacy rights, the present pictures were not the stimuli used in the experiment. The model in the sample pictures agreed to publish her pictures in the journal).

The stimuli were displayed against a gray background (11.95 cd/m2) on a 22-inch cathode-ray tube monitor (Philips 202P40, 100 Hz refresh rate, resolution of 1024 × 768 pixels). In addition, the monitor was gamma corrected according to Zhang *et al*.^23^. Participants were seated in a reclining chair in front of a computer screen in an electrically shielded and sound-attenuated room. They were instructed and familiarized with the experiment by a practice session. The experiment consisted of 280 trials including 9 conditions with 30 trials each. Ten practice trials preceded the test trials. The facial expressions judgment task was used, and participants were instructed to rapidly judge the facial expressions on the screen. Each trial consisted of the following sequence of events (see Fig. 1). At the beginning of each trial, a fixation cross appeared on the screen for 500 ms. Participants were instructed to fixate the cross. The fixation cross was followed immediately by a contextual sentence for 2000ms. After 300 ms into the presentation of the scene, the face appeared centrally on the screen for 500 ms. Participants were informed to concentrate on the face and categorize the facial expressions as either happy, neutral or sad by pressing corresponding words that were labeled on keys “F”, “Enter”, and “J”. The response keys were counterbalanced across participants. The inter-trial interval (ITI) was randomized between 600 and 800 ms.

#### Data analysis

For each participant in each condition, incorrect trials or trials with RTs beyond ± 2 *SD*s away from the mean and over 3000 ms were excluded from RT analysis^24^. These trials accounted for 5.60% of the total trials.

### Results

#### The language context effect of facial expression processing

Behavioral analyses were performed for RTs and ACCs (see Table 1). An ANOVA with repeated measurements was conducted to analyze RTs and ACCs with types of the language context (positive, neutral, negative) and types of facial expressions (positive, neutral, negative) as within-subject factors.

**Table 1:** RTs and ACCs for the judgment of facial expressions in different language contexts (*M* ± *SD*).

Language contexts	RTs (ms)			ACCs (%)		
	positive facial expressions	neutral facial expressions	negative facial expressions	positive facial expressions	neutral facial expressions	negative facial expressions
positive language context	417.40 ± 152.46	623.30 ± 263.93	651.80 ± 293.64	94.95 ± 10.18	85.16 ± 18.24	69.24 ± 18.05
neutral language context	460.93 ± 172.92	563.54 ± 211.63	638.55 ± 277.79	91.60 ± 12.23	88.49 ± 17.44	74.05 ± 18.54
negative language context	495.96 ± 247.08	609.17 ± 258.38	577.25 ± 243.13	85.76 ± 21.59	81.41 ± 20.01	79.29 ± 15.18

For the analysis of RTs, the main effect of types of facial expression was significant (*F*_(2,31)_ = 29.27, *p* < 0.001, η^2^_*p*_ = 0.65) indicating the RTs for judging positive facial expressions were significantly shorter than that of neutral (*p* < 0.05) and negative (*p* < 0.05) facial expressions while there was no significant difference in RTs between judging neutral and negative facial expressions (*p* = 0.202). For types of the language context, there was no significant difference (*F*_(2,31)_ = 0.66, *p* = 0.522). The interaction of types of the language context × types of facial expressions was significant (*F*_(4,29)_ = 4.68, *p* < 0.01, η^2^_*p*_ = 0.39). Further analysis showed that I) there was a significant difference in RTs for judging positive facial expressions in different language contexts (*F*_(2,31)_ = 5.65, *p* < 0.01, η^2^_*p*_ = 0.27) indicating that RTs in the positive language context were significantly shorter than that in neutral (*p* < 0.05) and negative (*p* < 0.05) language contexts, while there was no significant difference in RTs between neutral and negative language contexts (*p* = 0.388). II) There was a significant difference in RTs for judging neutral facial expressions in different language contexts (*F*_(2,31)_ = 5.42, *p* < 0.05, η^2^_*p*_ = 0.26) indicating that RTs in the neutral language context were significantly shorter than that in positive (*p* < 0.01) and negative language contexts (*p* = 0.061) while there was no significant difference in RTs between positive and negative language contexts (*p* = 0.659). III) There was a significant difference in RTs for judging negative facial expressions in different language contexts (*F*_(2,31)_ = 3.46, *p* < 0.05, η^2^_*p*_ = 0.18) indicating that the RTs in the negative language context were significantly shorter than that in positive (*p* < 0.05) and negative language contexts (*p* = 0.066) while there was no significant difference in RTs between positive and neutral language contexts (*p* = 0.800). The above results indicated that the RTs for judging facial expressions were influenced by the type of emotion of the language.

The analysis of the ACCs revealed that the main effect of the types of facial expression was significant (*F*_(2,31)_ = 36.04, *p* < 0.001, η^2^_*p*_ = 0.70). This indicated that the ACCs for judging positive facial expressions were significantly higher than those of neutral (*p* < 0.01) and negative (*p* < 0.01) facial expressions, and that the ACCs for judging neutral facial expressions were significantly higher than those of negative facial expressions (*p* < 0.01). There was no significant difference (*F*_(2,31)_ = 1.79, *p* = 0.183) in the main effect of the types of the language context. The interaction of the types of the language context × the types of facial expressions was significant (*F*_(4, 29)_ = 5.15, *p* < 0.01, η^2^_*p*_ = 0.42). Further analysis revealed the following. (I) There was a significant difference in the ACCs for judging positive facial expressions in different language contexts (*F*_(2,31)_ = 5.85, *p* < 0.01, η^2^_*p*_ = 0.27) indicating that the ACCs in the positive language context were significantly higher than those in the neutral (*p* < 0.05) and negative (*p* < 0.05) language contexts, while there was no significant difference in the ACCs between neutral and negative language contexts (*p* = 0.227). (II) There was a significant difference in the ACCs for judging neutral facial expressions in different language contexts (*F*_(2,31)_ = 5.26, *p* < 0.05, η^2^_*p*_ = 0.25) indicating that the ACCs in the neutral language context were significantly higher than that in the negative language context (*p* < 0.01) while there was no significant difference in the ACCs between positive and neutral language contexts (*p* = 0.169) as well as between positive and negative language contexts (*p* = 0.185). (III) There was a significant difference in the ACCs for judging negative facial expressions in different language contexts (*F*_(2,31)_ = 4.29, *p* < 0.05, η^2^_*p*_ = 0.22) indicating that the ACCs in the negative language context were significantly higher than that in the positive language context (*p* < 0.05) while there was no significant difference in the ACCs between positive and neutral language contexts (*p* = 0.221) as well as between neutral and negative language contexts (*p* = 0.200). The above results indicated that the ACCs for judging facial expressions were influenced by the types of emotion in the language.

#### The role of the language context effect

Previous studies^5,6^ show that the language context effect of emotional faces processing was mainly manifested in the processing speed (RTs) with the promotion when emotionally congruent and the inhibition when emotionally incongruent. A 2 (types of facial expressions: positive, negative) × 3 (emotional congruency: congruent, irrelevant, incongruent) repeated ANOVAs was adopted to the RTs. When the language context was emotionally congruent or incongruent with faces, we meant this condition as “congruent” or “incongruent” respectively. When the language context and faces were all neutral, we meant this condition as “irrelevant”. The main effect of the types of facial expressions was significant (*F*_(1,32)_ = 40.78, *p* < 0.001, η^2^_*p*_ = 0.56) indicating that the RTs for judging positive facial expressions (*M* = 458.10, *SD* = 190.33) were significantly shorter than that for judging negative facial expressions (*M* = 622.53, *SD* = 271.52; *p* < 0.001). The main effect of emotional congruency was also significant (*F*_(2,31)_ = 5.69, *p* < 0.01, η^2^_*p*_ = 0.27) indicating that the RTs for judging facial expressions when expressions and language contexts were emotionally congruent (*M* = 497.33, *SD* = 197.80) were significantly shorter than that of irrelevant (*M* = 549.74, *SD* = 225.36; *p* < 0.01) and incongruent (*M* = 573.88, *SD* = 270.36; *p* < 0.01) while there was no significant difference between irrelevant and incongruent (*p* = 0.150). The interaction of the types of facial expression × emotional congruency showed no significant difference (*F*_(1,31)_ = 0.42, *p* = 0.662). These results indicated that the language context effect on the facial expressions processing were mainly manifested as the promotion of emotional congruency.

Previous studies^5,6^, show that the language context effect on non-emotional (neutral) faces processing not only manifested in the processing speed (RTs), but also manifested in the judgment of types of facial expressions (ACCs). This effect mainly manifested as the inducement of the emotion of neutral faces. The judgment of the types of emotion of neutral faces would be induced by the emotional congruence of the context. There was a significant difference in judging neutral faces in different contexts (*F*_(2,31)_ = 5.42, *p* < 0.05, η^2^_*p*_ = 0.26) in the analysis of RTs, indicating that the RTs in the neutral language context (*M* = 563.54, *SD* = 211.63) were significantly shorter than those in the positive (*M* = 623.30, *SD* = 263.93; *p* < 0.05) and negative language contexts (*M* = 609.17, *SD* = 258.38; *p* < 0.05). There was no significant difference between the positive and the negative language contexts (*p* = 0.301). The analysis of ACCs showed a significant difference in judging neutral faces in different contexts (*F*_(2,31)_ = 5.26, *p* < 0.05, η^2^_*p*_ = 0.25) indicating that the ACCs in the neutral language context (*M* = 88.49, *SD* = 17.44) were significantly higher than those in the positive (*M* = 85.16, *SD* = 18.24; *p* = 0.060) and negative (*M* = 81.41, *SD* = 20.01; *p* < 0.01) language contexts. There was a marginally significant difference between positive and negative language contexts (*p* = 0.060).

We analyzed judgments when the participants mistakenly judged neutral facial expressions. A single factor repeated measure ANOVA was adopted and the judgments were divided into two parts including congruent and incongruent language contexts. Results indicated that there was a significant difference in different types of judgments (*F*_(1,32)_ = 16.22, *p* < 0.001, η^2^_*p*_ = 0.34) indicating that judgments congruent with the emotion of the language context (*M* = 3.71, *SD* = 4.33) were significantly higher than judgments incongruent with the emotion of the language context (*M* = 1.58, *SD* = 1.94; *p* < 0.001). It indicated that the language context effect on neutral faces processing was not only on the promotion when emotionally congruent and inhibition when emotionally incongruent, but also on the evoking effect on neutral faces. When participants mistakenly judged neutral facial expressions, they were more likely to judge congruent with the emotion of the language context, that is, judgments of the types of emotion of neutral faces were more inclined toward the congruent context.

### Brief summary

The results indicated that the language context affected facial expression processing. There was a promotion on facial expressions processing when the language context was emotionally congruent with faces. Moreover, the language context had an evoking effect on neutral faces. To be detailed, neutral facial expressions were evoked to be judged as positive in the positive language context while as negative in the negative language context. Although Experiment 1 found that the language context affected facial expression processing, it remained unclear that whether this effect was mandatory. In this way, this experiment investigated the processing characteristics of the language context effect on facial expression processing using the task-irrelevant paradigm (gender judgment task) to learn whether the language context effect on facial expression processing also had automated processing characteristics.

## Experiment 2

### Methods

#### Participants

Twenty-two right-handed college students (11 females) with a mean age of 22.08 (*SD* = 1.59) were paid to participate in the experiment. All participants had normal or corrected-to-normal vision. They reported no history of affective disorder and were free of any psychiatric medication. Each participant signed an informed consent form prior to the experiment. The study was approved by the Ethics Committee of Ningbo University in accordance with the Declaration of Helsinki.

#### Experimental design

A 3 (types of the language context: positive, neutral, negative) × 3 (types of facial expressions: positive, neutral, negative) within-subject design was adopted. The dependent variables were the ACCs for judging the gender of faces and the peak values of N170 components as well as average amplitude of LPP component. Since this experiment used the task-irrelevant paradigm, the RTs for judging the gender of the faces were used only for screening out frivolous participants. The ACCs for judging the gender of faces in this experiment were all above 80%.

#### Stimuli

All the stimuli used in this experiment were the same as those in Experiment 1.

#### Procedure

**Figure 2:**
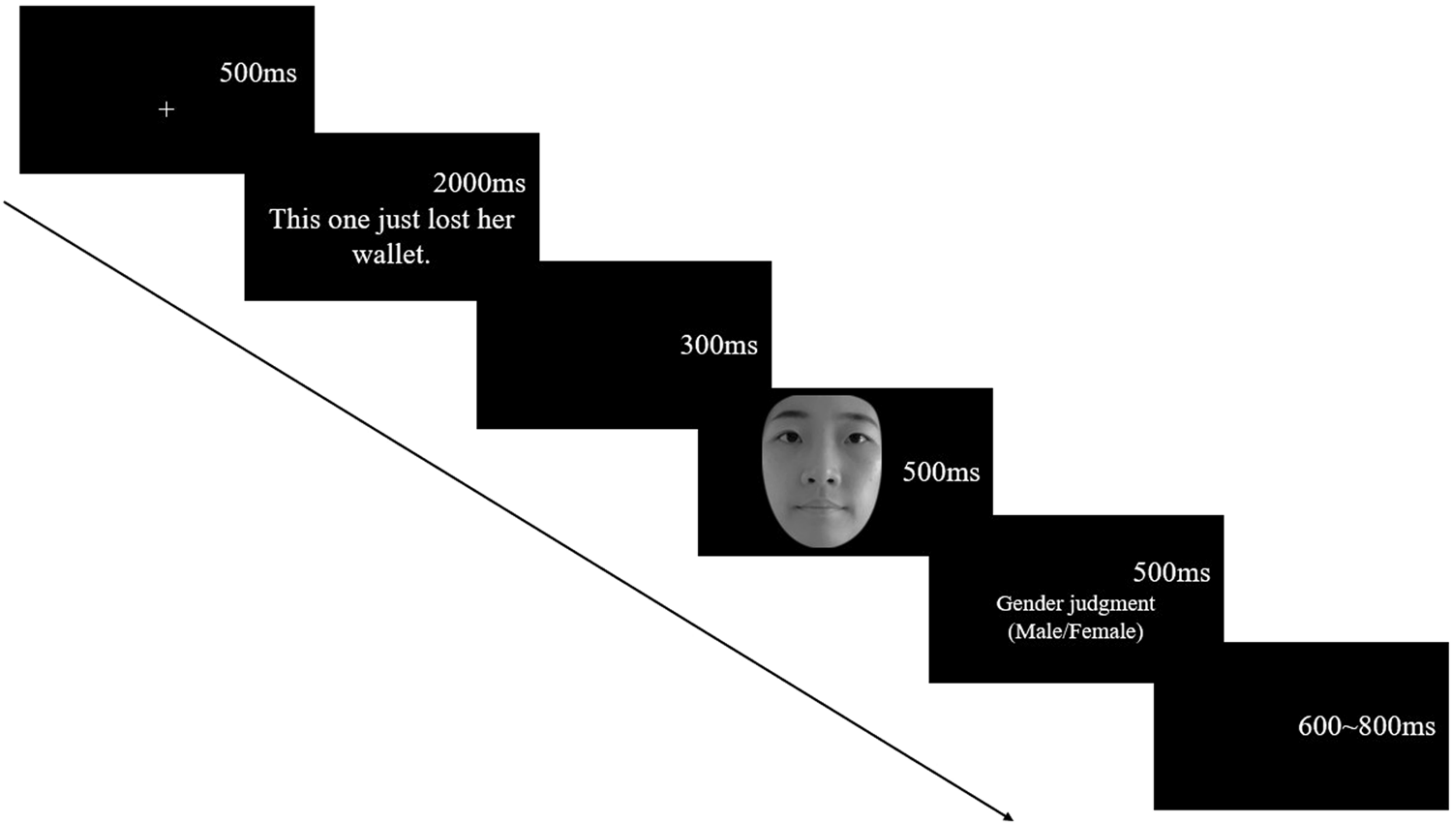
Timings and displays of one trial in the task. (Please reader note that due to the privacy rights, the present pictures were not the stimuli used in the experiment. The model in the sample pictures agreed to publish her pictures in the journal).

The stimuli were displayed against a gray background (11.95 cd/m2) on a 22-inch cathode-ray tube monitor (Philips 202P40, 100 Hz refresh rate, resolution of 1024 × 768 pixels). In addition, the monitor was gamma corrected according to Zhang *et al*.^23^. Participants were seated in a reclining chair in front of the computer screen in an electrically shielded and sound-attenuated room. They were instructed on and familiarized with the experiment through a practice session. The experiment consisted of 550 trials comprising 9 conditions with 60 trials each, and 10 practice trials preceding the test trials. The gender judgment task was used. The participants were instructed to rapidly judge the gender of the faces on the screen. Each trial consisted of the following sequence of events (Fig. 2). At the beginning of each trial, a fixation cross appeared on screen for 500 ms. Participants were instructed to fixate the cross. The fixation cross was followed immediately by a contextual sentence for 2000ms. Then, a black screen appeared for 300 ms and then facial expressions appeared for 500 ms. Participants were told to concentrate on the face and judged the gender of the face by pressing the corresponding buttons (“F” and “J” labeled with words “male” and “female”). The response keys were counterbalanced across participants. The inter-trial interval (ITI) varied randomly between 600 and 800 ms.

#### Electrophysiological recording and analysis

The electroencephalogram (EEG) output was continuously recorded using an electrode cap with 64 Ag/AgCl electrodes mounted according to the extended international 10–20 system. Horizontal electrooculogram (HEOG) and vertical electrooculogram (VEOG) were recorded with two pairs of electrodes: one pair placed 10 mm from lateral canthi of both eyes, and the other pair placed on the left infraorbital and supraorbital areas. Both EEG and EOG were amplified and digitized by a Neuroscan Synamp^2^ Amplifier with a band pass of 0.05–100 Hz and a sampling rate of 500 Hz. The tip of the nose served as reference during the recording. Electrode impedance was maintained below 5kΩ throughout the experiment.

**Figure 3:**
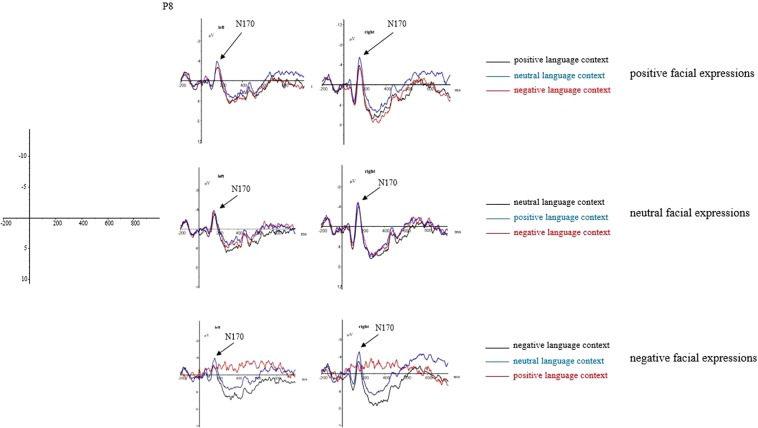
The grand-average ERPs elicited by positive, neutral, and negative facial expressions in different language contexts at P8 site.

**Figure 4:**
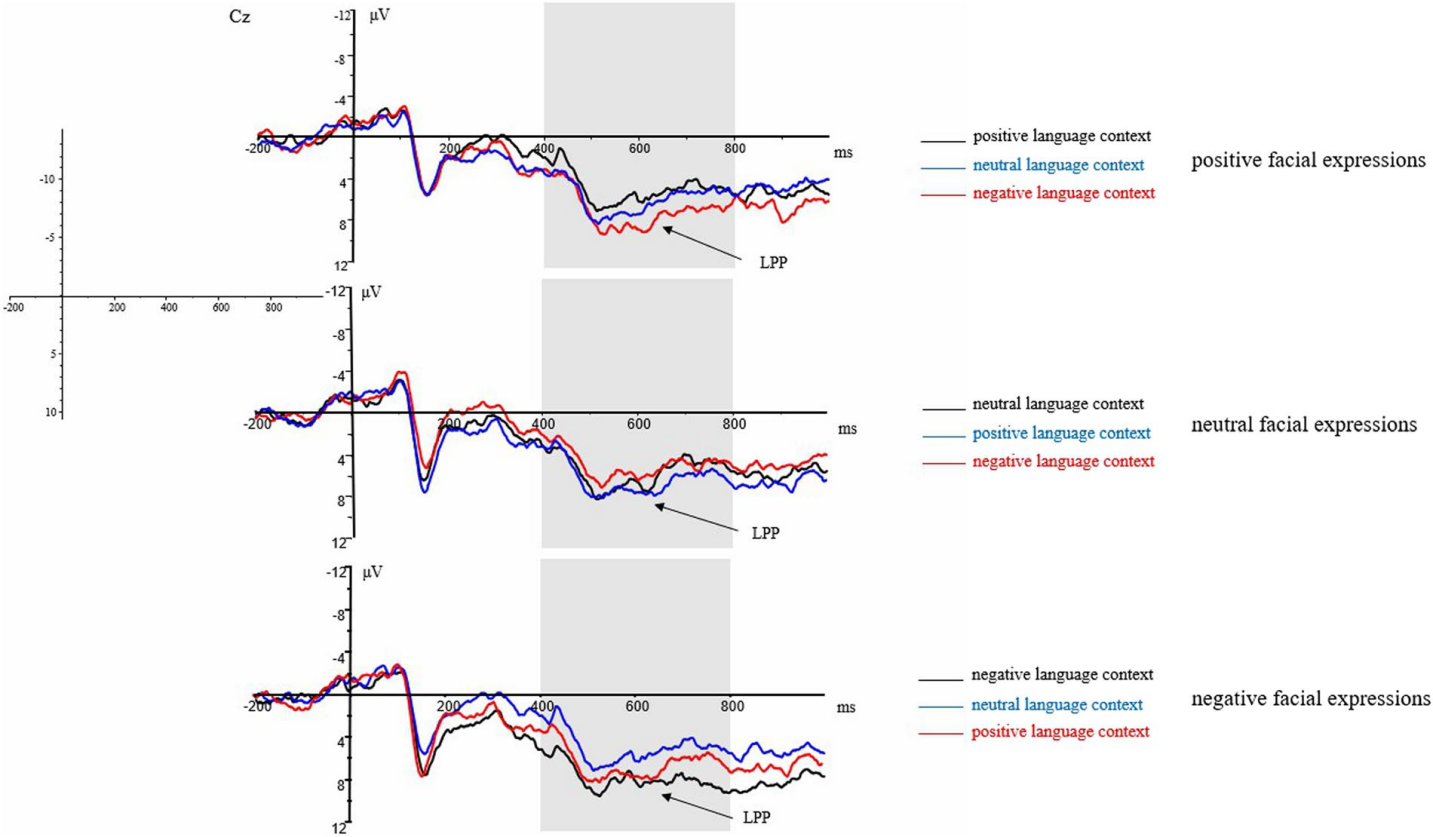
The grand-average ERPs elicited by positive, neutral, and negative facial expressions in different language contexts at Cz site.

**Figure 5:**
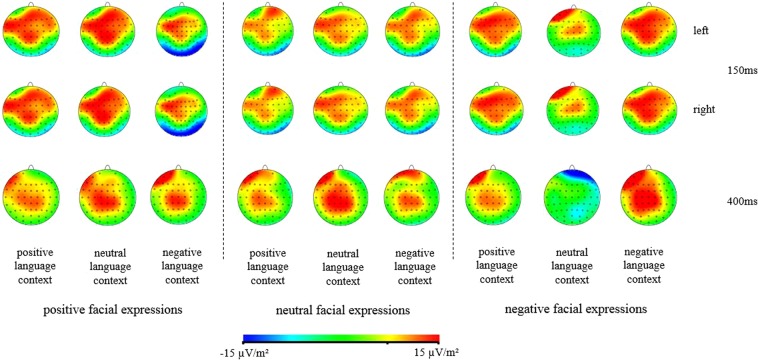
The 2D scalp topographic distribution of the LPP component elicited by positive, neutral, and negative facial expressions in different language contexts.

The averaged ERPs were digitally filtered with a low-pass filter at 30 Hz (24 dB/octave). The EOG artifacts were corrected offline using a regression-based procedure^25^. The EEG was segmented into the epoch from −200 ms to 800 ms in which 0 ms was the time the face was shown (time-locked to faces onset). According to previous studies^26–28^, trials contaminated by amplifier clipping, bursts of electromyographic activity, or peak-to-peak deflection exceeding ±100 μV were excluded from averaging. Generally speaking, the criteria for removing participants is about 10% artifacts of trials^26^. According to this criterion, data of five participants were removed for the EEG artifacts and 17 valid participants (9 males and 8 females) were reserved. The statistical analysis was based on within-subject factorial models in which the peak amplitudes (relative to the pre-stimulus baseline; N170) and the mean amplitudes (LPP) of original ERP components were dependent variables. The measurement windows were determined by the visual inspection of grand-average waveforms (130–200 ms for N170; see Figs 3, 4 and 5). The mean amplitudes were measured at the 400–800 ms time windows for LPP. For N170 (P7, P8, PO7, PO8) components, four electrode sites were analyzed. P7 and PO7 were for the left hemisphere, while P8 and PO8 were for the right hemisphere. Based on the LPP scalp distribution, nine electrode sites (F1, Fz, F2, C1, Cz, C2, P1, Pz, P2) were selected for measurement. Peak and mean amplitudes were assessed via ANOVAs with repeated measurements. The *p*-value was corrected using the Greenhouse–Geisser epsilon.

The analysis of the behavioral data in the current experiment was the same as in Experiment 1. Trials removed in the current experiment accounted for 4.53% of the total trials.

### Results

#### Behavioral performance

Behavioral analyses were performed for RTs and ACC (see Table 2). An ANOVA with repeated measurements was conducted to analyze RTs and ACCs with types of the language context (positive, neutral, negative) and types of facial expressions (positive, neutral, negative) as within-subject factors. For the ANOVAs of RTs and ACCs, the main effect of types of facial expressions was significant (*F*_(2,20)_ = 10.59, *p* < 0.01, η^2^_*p*_ = 0.51 and *F*_(2,20)_ = 53.49, *p* < 0.001, η^2^_*p*_ = 0.84, respectively), while those of types of the language context (*F*_(2,20)_ = 0.18, *p* = 0.837 and *F*_(2,20)_ = 0.69, *p* = 0.512, respectively) and the interaction of types of the language context × types of facial expressions (*F*_(4, 18)_ = 0.21, *p* = 0.928 and *F*_(4, 18)_ = 0.65, *p* = 0.634, respectively) were non-significant.

**Table 2:** RTs and ACCs for judgment of gender of faces in different language contexts (*M* ± *SD*).

Language contexts	RTs (ms)			ACCs (%)		
	positive facial expressions	neutral facial expressions	negative facial expressions	positive facial expressions	neutral facial expressions	negative facial expressions
positive language context	232.18 ± 57.44	248.18 ± 60.83	231.18 ± 53.61	96.44 ± 3.65	91.21 ± 4.75	96.21 ± 3.57
neutral language context	234.77 ± 61.27	247.69 ± 58.25	235.32 ± 52.72	96.06 ± 5.69	91.21 ± 5.52	96.52 ± 3.71
negative language context	234.24 ± 56.17	245.90 ± 62.48	236.05 ± 53.98	94.62 ± 6.13	91.67 ± 4.02	96.51 ± 3.04

#### ERPs

The grand average ERP waveforms of the relevant components evoked by positive, neutral, and negative facial expressions in different language contexts are presented in Figs 3, 4 and 5.

N170 component (130~200 ms): The repeated measure ANOVAs with types of facial expressions (positive, neutral, negative), types of the language context (positive, neutral, negative), and types of hemispheres (left, right) were conducted to analyze the peak amplitudes of N170.

The main effect of types of hemispheres was significant (*F*_(1,16)_ = 30.73, *p* < 0.001, η^2^_*p*_ = 0.658), indicating a significantly larger N170 at left hemisphere (*M* = −1.41 μV, *SD* = 0.92) than right hemisphere (*M* = −5.43 μV, *SD* = 1.00). While the main effects of types of facial expressions (*F*_(1,16)_ = 0.03, *p* = 0.866) and types of the language context (*F*_(1,16)_ = 3.37, *p* = 0.085) were of no significant difference. Moreover, the interaction of types of the language context × types of facial expressions (*F*_(1,16)_ = 38.88, *p* < 0.001, η^2^_*p*_ = 0.708) and the interaction of types of hemispheres × types of the language context × types of facial expressions (*F*_(1,16)_ = 9.13, *p* < 0.01, η^2^_*p*_ = 0.363) were significant. While the interaction of types of hemispheres × types of the language context (*F*_(1,16)_ = 0.21, *p* = 0.654) and the interaction of types of hemispheres × types of facial expressions (*F*_(1,16)_ = 1.23, *p* = 0.284) were all of no significant difference.

Further analysis regarding the significant interaction of types of the language context × types of facial expressions indicated that I) Amplitudes of positive facial expressions in the positive language context (*M* = −3.19 μV, *SD* = 0.902) were larger than those in the neutral (*M* = −3.25 μV, *SD* = 0.843; *p* = 0.541) and negative (*M* = −3.87 μV, *SD* = 0.922; *p* = 0.062) language contexts without significant difference. In addition, there was no significant difference of amplitudes of positive facial expressions between neutral and negative language contexts (*p* = 0.421). II) Amplitudes of neutral facial expressions in the neutral language context (*M* = −3.33 μV, *SD* = 0.90) were larger than those in the positive (*M* = −3.67 μV, *SD* = 0.96; *p* = 1.00) and negative (*M* = −3.40 μV, *SD* = 0.98; *p* = 0.756) language contexts without significant difference. In addition, there was no significant difference of amplitudes of neutral facial expressions between positive and negative language contexts (*p* = 0.887). III) Amplitudes of neutral facial expressions in the negative language context (*M* = −2.96 μV, *SD* = 1.06) were larger than those in the positive (*M* = −4.11 μV, *SD* = 0.94; *p* = 0.455) and negative (*M* = −3.06 μV, *SD* = 0.84; *p* = 0.483) language contexts without significant difference. In addition, there was no significant difference of amplitudes of negative facial expressions between positive and negative language contexts (*p* = 0.143).

LPP component (400~800 ms): The repeated measure ANOVAs with types of facial expressions (positive, neutral, negative), types of the language context (positive, neutral, negative), and types of hemispheres (left, right) were conducted to analyze the mean amplitudes of LPP.

The main effects of types of facial expressions (*F*_(1,15)_ = 0.33, *p* = 0.576), types of the language context (*F*_(1,15)_ = 0.25, *p* = 0.624) and types of hemispheres (*F*_(1,15)_ = 0.12, *p* = 0.735) were all of no significant difference. The interaction of types of the language context × types of facial expressions was significant (*F*_(1,15)_ = 9.27, *p* < 0.01, η^2^_*p*_ = 0.382). While the interaction of types of hemispheres × types of the language context (*F*_(1,15)_ = 0.07, *p* = 0.795), the interaction of types of hemispheres × types of facial expressions (*F*_(1,15)_ = 0.09, *p* = 0.773) and the interaction of types of hemispheres × types of the language context × types of facial expressions (*F*_(1,15)_ = 0.09, *p* = 0.772) were all of no significant difference.

Further analysis regarding the significant interaction of types of the language context × types of facial expressions indicated that I) Amplitudes of negative facial expressions (*M* = 6.60 μV, *SD* = 1.44) were significantly larger than that of positive facial expression (*M* = 5.47 μV, *SD* = 1.37; *p* < 0.05) but not neutral facial expressions (*M* = 5.95 μV, *SD* = 1.55; *p* = 0.527) in the positive language context. While there was no significant difference of amplitudes between positive and neutral facial expressions (*p* = 0.763) in the positive language context. II) Amplitudes of positive facial expressions (*M* = 6.60 μV, *SD* = 1.34) were larger than that of neutral (*M* = 5.74 μV, *SD* = 1.37; *p* = 0.450) and negative (*M* = 5.34 μV, *SD* = 1.35; *p* = 0.239) facial expressions without significant difference in the neutral language context. In addition, there was no significant difference of amplitudes between neutral and negative facial expressions (*p* = 0.869) in the neutral language context. II) Amplitudes of neutral facial expressions (*M* = 6.31 μV, *SD* = 1.49) were larger than that of positive (*M* = 6.30 μV, *SD* = 1.45; *p* = 1.00) and negative (*M* = 5.76 μV, *SD* = 1.59; *p* = 0.501) facial expressions without significant difference in the negative language context. In addition, there was no significant difference of amplitudes between positive and negative facial expressions (*p* = 0.641) in the neutral language context.

### Brief summary

Results indicated that the language context affected facial expressions processing. When the language context and facial expressions were emotionally incongruent, larger amplitudes of N170 and LPP appeared, indicating inhibition during emotional incongruence. Thus, the language context effect on facial expression processing was mandatory. In other words, the language context effect was an unintentional automated processing.

## Discussion

The current study mainly investigated the language context effect on facial expression processing; the findings revealed that the characteristics of automated processing of the language context effect on facial expression processing were mandatory.

Experiment 1 used the classic facial expression categorization task to investigate the language context effect of facial expression processing and its specific performance. Results revealed that the RTs and ACCs for judging emotional faces (happy or sad facial expressions) and non-emotional faces (neutral faces) were all influenced by the emotion of the language context. This indicated that judgments of facial expressions were influenced by the language context, regardless of the emotion on the face. The language context effect on emotional faces processing was mainly manifested in the promotion when emotionally congruent. When the language context and facial expressions were emotionally congruent, individuals’ processing of facial expressions was faster and that in the ACCs were higher. When the emotional value carried by the background information was consistent with the emotional meaning expressed by the facial expressions, it would promote the facial expression processing. Moreover, previous studies on the language context effect also found that this consistency effect was reflected not only in the RTs and ACCs but also in individuals’ preferences for the faces^3^. This suggested that the language context effect was manifested in not only simple cognitive responses but also deeper emotional assessments that required integration of prior experience. In addition, the language context effect on non-emotional faces processing was not only manifested in the promotion when emotionally congruent and the inhibition when emotionally incongruent of the processing rate of neutral faces, but also evoked effect on neutral faces. That is, neutral faces in the negative language context were more likely to be recognized as negative facial expressions, while neutral faces in the positive language context were more likely to be recognized as positive facial expressions. This result was also consistent with those of previous studies. Emotional language contexts can provide a certain emotional color to neutral faces, and the emotional meaning given is also consistent with that of the language context itself^10,11^. In addition to the language context, some studies showed that other contextual information (e.g., scenes, sounds) could also have emotionally evoking effects on neutral faces and the evoking or coupling effects of such emotions could occur in the early perceptual coding stage of face processing^9,14^. Wieser and Brosch believed that neutral faces processing was susceptible to the language context^12^. It might be that the emotional meaning expressed by neutral faces was vague. When the configuration information of such face stimuli could not be clearly expressed by their emotional states, individual judged the emotional states according to the surroundings of faces.

Experiment 2 used the gender judgment tasks unrelated to facial expression processing, and combined it with event-related potentials techniques to investigate whether the language context effect was mandatory. Results revealed that in the gender judgment task, the facial expression processing was still influenced by the language context, indicating that the language context effect still existed without intention. No language context effect was observed in the behavioral data in this experiment where the emotion-irrelevant task was used. Since this experiment used the task-irrelevant paradigm, the RTs for judging the gender of the faces were used only for screening out frivolous participants and the ACCs for judging the gender of faces in this experiment were all above 80%. This was just the effectiveness of the emotion-irrelevant manipulation in this experiment. The language context can automatically affect the early perceptual coding and late processing stages of facial expression processing. Furthermore, there were larger amplitudes of N170 and LPP when facial expressions were incongruent with emotions of the language context and neutral faces in the negative language context could activate larger amplitude of LPP. In face studies, the N170 is an important early ERP component related with to processing, which reflects the structured coding of faces^29^. Therefore, results of this experiment showed that the language context could automatically affect the structured coding of facial expressions without intention, showing the inhibition of facial expression processing when emotional conflicts disturb the structured coding of faces. In emotional studies, the LPP component reflects the advanced processing of the cerebral cortex for sustained attention, emotional evaluation, and elaborate processing of emotional stimuli^30^. Therefore, results of this experiment indicated that the language context could automatically influence the high-level cognitive processing of facial expressions without intention. When expressing emotional conflicts, the facial expressions processing occupies more attention resources, allowing individuals to perform a more detailed processing of the emotional information of faces. The above results were also consistent with those of the previous studies on language contexts. For example, Diéguez-Risco *et al*. found that larger amplitudes of N170 and LPP were activated when the language context was incongruent with emotions of faces to affect the early structural coding of face processing and later emotion assessment^4^. Moreover, they also found that neutral faces in the negative language context could elicit larger amplitude of LPP^4^. This may be because compared to other language contexts, negative information in the negative language context has higher prominence, which provides more interference for the cognitive processing of the later faces presented to make participants invest more attention resources in elaborate processing.

The current study had the following significance. One the one hand, it broadens research on the automatic characteristics of the scene effect to a certain extent, and explores whether this automated processing can occur at the level of not only perceptual processing but also deeper semantic processing. It contributes to answering whether the automatic characteristics of the scene effect had certain universality. On the other hand, the automated processing characteristics of the language context effect revealed by the current study can provide evidence for assessing the important position of the language context when individuals process facial expressions in real life interactions and investigate special groups with emotional communication barriers (e.g., autism, social phobia, and schizophrenia) in the future research.

The limitations to the current study that constrained the interpretation of the findings are as follows. First, the current study only focused on the language context effect on happy, sad, and neutral facial expressions processing; other negative facial expressions, like scared, angry, disgusting, were not investigated. Future studies can expand emotional categories to investigate not only basic emotions but also other complex emotions, such as shyness and jealousy. Second, due to the particularity of contextual materials, it is not suitable to use some classic paradigms, such as emotional Stroop paradigm and subliminal emotional initiation, to explore the unconsciousness of the language context. Future studies should find a suitable paradigm to echo the above issues and provide empirical evidence for whether the language context effect was mandatory. Third, the current study only explored the autonomic characteristics of the language context effect from the time course of facial expression processing and perception judgment. Future studies can combine the functional magnetic resonance imaging (fMRI) to further explore the cognitive neural mechanism of this automated processing.
